# The Differential Effect of a Shortage of Thyroid Hormone Compared with Knockout of Thyroid Hormone Transporters Mct8 and Mct10 on Murine Macrophage Polarization

**DOI:** 10.3390/ijms25042111

**Published:** 2024-02-09

**Authors:** Esmée Hoen, Franka M. Goossens, Kim Falize, Steffen Mayerl, Anne H. van der Spek, Anita Boelen

**Affiliations:** 1 Endocrine Laboratory, Department of Laboratory Medicine, Amsterdam Gastroenterology Endocrinology & Metabolism (AGEM), Amsterdam UMC, University of Amsterdam, 1105 AZ Amsterdam, The Netherlands; e.hoen@amsterdamumc.nl (E.H.); k.f.falize@amsterdamumc.nl (K.F.); 2 Department of Endocrinology, Diabetes & Metabolism, University Duisburg-Essen, 47057 Essen, Germany; 3 Department of Endocrinology and Metabolism, Amsterdam Gastroenterology Endocrinology & Metabolism (AGEM), Amsterdam UMC, University of Amsterdam, 1105 AZ Amsterdam, The Netherlands

**Keywords:** Mct8, Mct10, thyroid hormone, T3, bone marrow-derived macrophages, thyroid hormone transporters, innate immune system

## Abstract

Innate immune cells, including macrophages, are functionally affected by thyroid hormone (TH). Macrophages can undergo phenotypical alterations, shifting between proinflammatory (M1) and immunomodulatory (M2) profiles. Cellular TH concentrations are, among others, determined by TH transporters. To study the effect of TH and TH transporters on macrophage polarization, specific proinflammatory and immunomodulatory markers were analyzed in bone marrow-derived macrophages (BMDMs) depleted of triiodothyronine (T3) and BMDMs with a knockout (KO) of Mct8 and Mct10 and a double KO (dKO) of Mct10/Mct8. Our findings show that T3 is important for M1 polarization, while a lack of T3 stimulates M2 polarization. Mct8 KO BMDMs are unaffected in their T3 responsiveness, but exhibit slight alterations in M2 polarization, while Mct10 KO BMDMs show reduced T3 responsiveness, but unaltered polarization markers. KO of both the Mct8 and Mct10 transporters decreased T3 availability and, contrary to the T3-depleted BMDMs, showed partially increased M1 markers and unaltered M2 markers. These data suggest a role for TH transporters besides transport of TH in BMDMs. This study highlights the complex role of TH transporters in macrophages and provides a new angle on the interaction between the endocrine and immune systems.

## 1. Introduction

Thyroid hormones (THs) are important players in gene regulation and are essential for development, growth and energy metabolism [1,2]. In recent years it has become clear that THs are also indispensable for optimal macrophage function [3,4,5,6]. Macrophages are mononuclear phagocytic cells that belong to the innate immune system and are crucial for both host defense against infection and tissue homeostasis [7]. Macrophages can be tissue-specific, proliferating locally, or derived from circulating monocytes that infiltrate the inflamed tissue where they subsequently differentiate into macrophages [8,9,10]. Macrophages are capable of polarization, a process in which they adopt a different phenotype based on signals from their environment. These phenotypes range along a spectrum from proinflammatory to immunomodulatory. The outer ends of that spectrum are the so-called M1 and M2 macrophages [11,12]. M1 macrophages are proinflammatory macrophages responsible for microbial killing and eliciting an immune response [13]. These macrophages express surface proteins, such as CD80 and CD86, express proinflammatory genes, including *IL-1b*, *CD38*, formyl peptide receptor 2 (*Fpr2*) and G-protein-coupled receptor 18 (*Gpr18*), and secrete proinflammatory cytokines [14,15,16]. M2 macrophages exert more immunomodulatory effects and have homeostatic properties, such as tissue repair and remodeling [13]. M2 macrophages express the surface protein CD206 and immunomodulatory genes, such as early growth response 2 (*Egr2*), arginase 1 (*Arg1*), *IL-10* and *c-Myc*, and secrete anti-inflammatory cytokines [14,15,16]. Dysfunction of proper and adequate macrophage polarization is associated with autoimmune, metabolic and neurodegenerative diseases, cancer and other pathologies [7,17,18]. Interestingly, recent studies indicate that TH can influence polarization of macrophages [3,4,5].

Multiple studies have reported on the in vitro effect of TH on macrophage polarization. Stimulation of murine bone marrow-derived macrophages (BMDMs) with supraphysiological levels of the active form of thyroid hormone, triiodothyronine (T3), induces M1 polarization and attenuates M2 polarization [19]. Moreover, a murine macrophage cell line produces more nitric oxide, a hallmark of M1 polarization, when stimulated with T3 or the prohormone thyroxine (T4) [20]. This effect appears to be mediated through intracellular T3 signaling as knockdown of TRα, the dominant TH receptor (TR) in macrophages, resulting in inhibited M1 and increased M2 polarization gene markers [21]. 

In order for TH to exert its action, it needs to be taken up by the cell, making cellular transport of TH one of the crucial determinants of intracellular TH availability. The monocarboxylate transporters 8 (Mct8) and 10 (Mct10) are known to be present on macrophages, although Mct10 is the more prominent transporter [21]. The Mct8 transporter is involved in the influx and efflux of both T3 and T4, whereas Mct10 preferentially transports T3 in a bidirectional manner and not T4 [22,23,24]. Furthermore, Mct10 is also known to transport aromatic amino acids across the cell membrane, whereas transport of molecules other than TH metabolites by Mct8 is not described to our knowledge [22,23,24]. Mouse models of Mct8 knockout (KO), Mct10 KO and Mct10/Mct8 double KO (dKO) have been established to study these transporters [25]. Mct8 KO mice show elevated serum T3 and low serum T4, while Mct10 KO mice present normal serum TH concentrations and Mct10/Mct8 dKO mice are reported to show increased serum T3 levels and normal serum T4 [25].

Other TH transporters exist, although their presence on macrophages has not been reported [22,23,24]. L-type amino acid transporters 1 (Lat1) and 2 (Lat2) are known to transport both neutral amino acids and T3 into the cell, while Lat1 also transports T4 to a lesser extent than T3. Lat1 and Lat2 are not responsible for the efflux of T3 or T4 [24].

In this report, we aim to investigate the role of T3 transport by the TH transporters Mct8 and Mct10 on macrophage function. To this end, we studied the capacity of BMDMs derived from mice with a KO of the Mct8 (Mct8 KO) or Mct10 (Mct10 KO) transporter or dKO of both transporters (Mct10/Mct8 dKO) to polarize toward an M1 or M2 phenotype.

## 2. Results

### 2.1. Transporter Expression in the KO BMDMs

**Figure 1:**
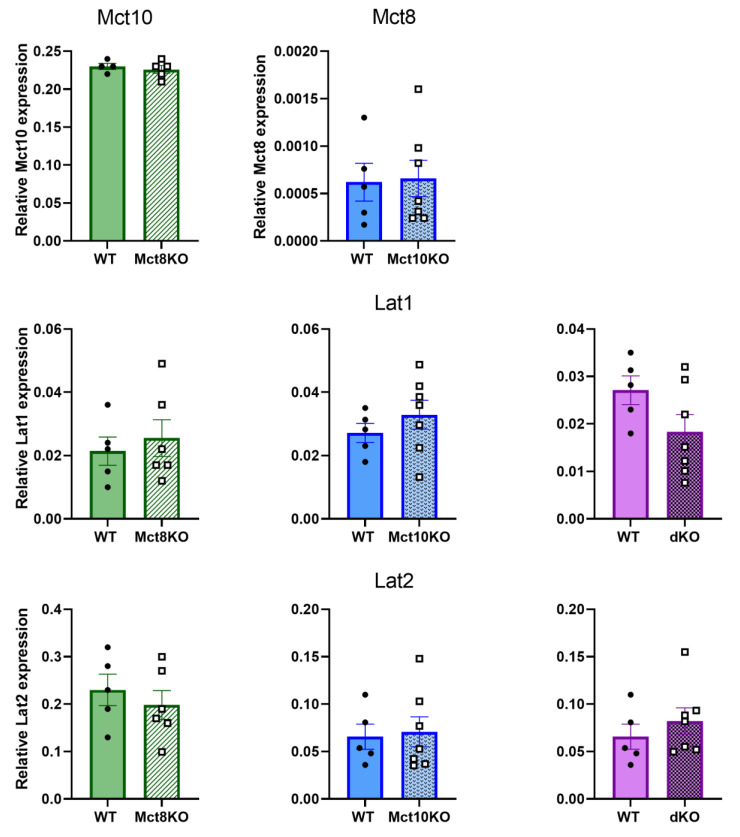
Relative mRNA expression of Mct8, Mct10, Lat1 and Lat2 in WT, Mct8 KO, Mct10 KO and Mct10/Mct8 dKO (dKO) BMDMs. Colored bars with closed circles represent WT BMDMs and patterned bars with open squares represent Mct8 KO, Mct10 KO and Mct10/Mct8 dKO BMDMs. Mean values ± SEM are depicted. Differences between groups were analyzed using unpaired two-tailed Student’s *t*-test.

To confirm the purity of our macrophage population, we assessed the percentage of CD11b/F4/80+ cells, which was always at least 83% of the total population. Moreover, no difference in the percentage of CD11b+/F4/80+ cells was observed between genotypes (Figure S1). To determine whether TH transporters are differentially expressed in the KO models as compensation for the loss of the transporters, we measured mRNA expression of Mct10 in the Mct8 KO and Mct8 in Mct10 KO macrophages. Additionally, we measured mRNA expression of the transporters Lat1 and Lat2 in all KO models. None of the transporters showed altered expression in any of the KO models in all M0 BMDMs compared with wild-type (WT) BMDMs (Figure 1). This was repeated in M1 and M2 BMDMs derived from KO mice where expression of the transporters was unaltered as well (Figures S2 and S3). The fact that Lat1 and Lat2 were expressed on BMDMs suggests that these transporters are present on murine macrophages and possibly contribute to TH transport in these cells. 

### 2.2. T3-Responsive Gene Expression in BMDMs

**Figure 2:**
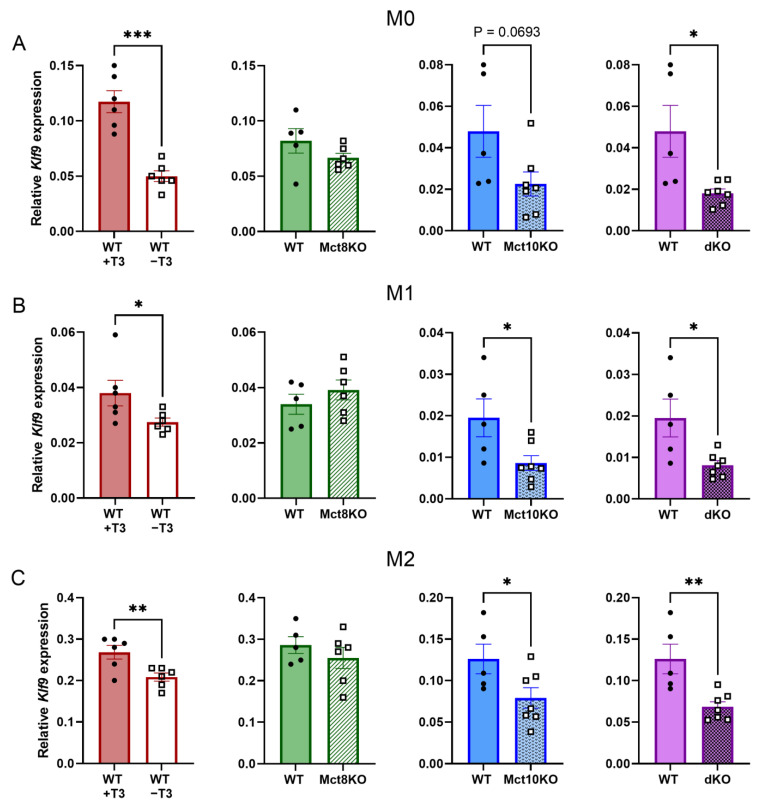
Relative *Klf9* mRNA expression in WT, Mct8 KO, Mct10 KO and Mct10/Mct8 dKO (dKO) M0 (**A**) M1 (**B**) and M2 (**C**) BMDMs. Colored bars with closed circles represent WT BMDMs treated with 10 nM T3 or WT BMDMs and open or patterned bars with open squares represent WT BMDMs treated with T3-depleted medium or Mct8 KO, Mct10 KO and Mct10/Mct8 dKO BMDMs. Mean values ± SEM are depicted. Differences between groups were analyzed using paired (T3-depleted or stimulated BMDMs) or unpaired (WT, Mct8 KO, Mct10 KO and Mct10/Mct8 dKO BMDMs) two-tailed Student’s *t*-test: * *p* < 0.05, ** *p* < 0.01, *** *p* < 0.001.

Expression of Krueppel-like factor 9 (*Klf9*), a positively T3-regulated gene, decreased in WT M0 BMDMs without T3 compared with WT M0 BMDMs that were cultured in the presence of T3, as expected (Figure 2A). No change in *Klf9* expression was observed in BMDMs derived from Mct8 KO mice, compared with WT BMDMs, while *Klf9* expression showed a downward trend (*p* = 0.069) in Mct10 KO BMDMs and decreased significantly in BMDMs derived from Mct10/Mct8 dKO mice (Figure 2A). In M1- and M2-polarized macrophages, *Klf9* expression was also unaffected by Mct8 KO; however, both Mct10 KO and Mct10/Mct8 dKO resulted in decreased *Klf9* expression (Figure 2B,C). These results suggest that T3 availability is reduced in both M1 and M2 Mct10 KO and Mct10/Mct8 dKO BMDMs, but not in Mct8 KO BMDM, implying that Mct10 is the main thyroid hormone transporter in murine BMDMs.

### 2.3. M1 Membrane Marker Expression of CD80 and CD86 in BMDMs

**Figure 3:**
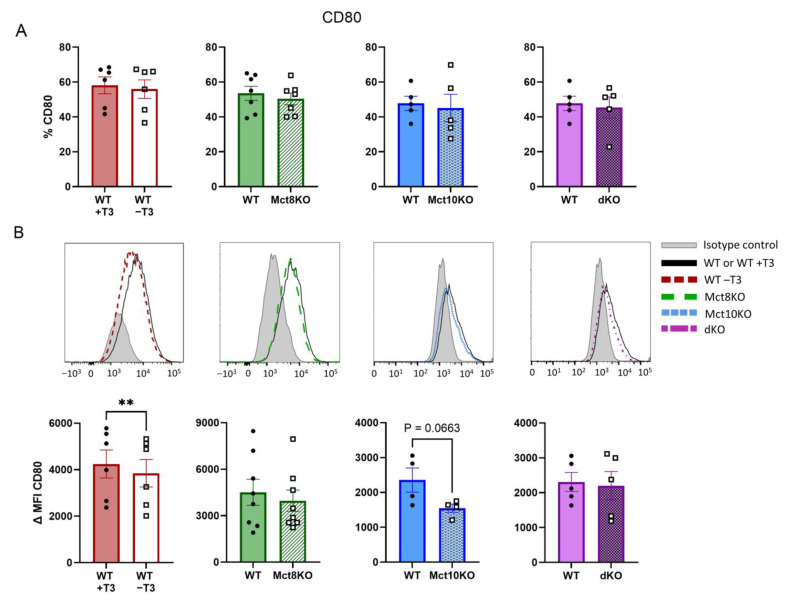
Expression of proinflammatory surface marker CD80 as measured by flow cytometry in M1-polarized WT, Mct8 KO, Mct10 KO and Mct10/Mct8 dKO (dKO) BMDMs. Colored bars with closed circles represent WT BMDMs treated with 10 nM T3 or WT BMDMs and open or patterned bars with open squares represent WT BMDMs treated with T3-depleted medium or Mct8 KO, Mct10 KO and Mct10/Mct8 dKO BMDMs. The percentage of cells expressing CD80 (**A**) and representative histograms with accompanying quantified delta median fluorescence intensities (ΔMFIs, **B**) are shown. Mean values ± SEM are depicted. Differences between groups were analyzed using a paired two-tailed Student’s *t*-test: ** *p* < 0.01.

Macrophages polarized toward the M1 phenotype are characterized by cell surface expression of specific markers, including CD80 and CD86 [15,16]. The expression of these markers was determined by flow cytometry. The percentage of cells expressing CD80 remained unchanged in all M1 BMDMs (Figure 3A). Expression levels of CD80, however, significantly decreased in BMDMs depleted of T3, while expression of this marker was unaltered in all the transporter KOs, except for a downward trend in the Mct10 KO BMDMs (*p* = 0.066) (Figure 3B). 

**Figure 4:**
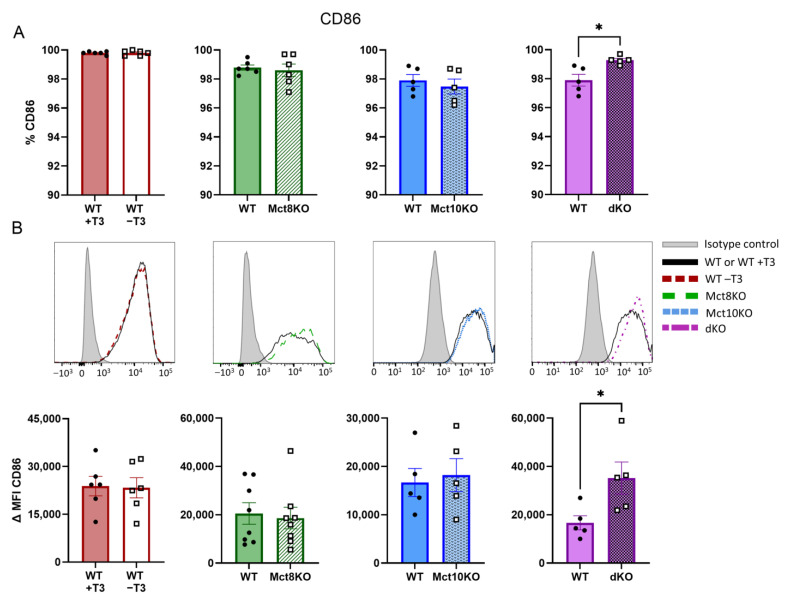
Expression of proinflammatory surface marker CD86 as measured by flow cytometry in M1-polarized WT, Mct8 KO, Mct10 KO and Mct10/Mct8 dKO (dKO) BMDMs. Colored bars with closed circles represent WT BMDMs treated with 10 nM T3 or WT BMDMs and open or patterned bars with open squares represent WT BMDMs treated with T3-depleted medium or Mct8 KO, Mct10 KO and Mct10/Mct8 dKO BMDMs. The percentage of cells expressing CD86 (**A**) and representative histograms with accompanying quantified delta median fluorescence intensities (ΔMFIs, **B**) are shown. Mean values ± SEM are depicted. Differences between groups were analyzed using a paired two-tailed Student’s *t*-test: * *p* < 0.05.

CD86 expression was not affected in the T3-depleted or the single transporter KO M1 BMDMs. Conversely, CD86 expression increased significantly in M1 BMDMs of Mct10/Mct8 dKO mice compared with those of WT mice (Figure 4A,B). These observations suggest that depletion of T3 in murine macrophages partially inhibits M1 marker expression, while a simultaneous lack of Mct8 and Mct10 partially enhances this.

### 2.4. M1 mRNA Expression and Cytokine Secretion in BMDMs

**Figure 5:**
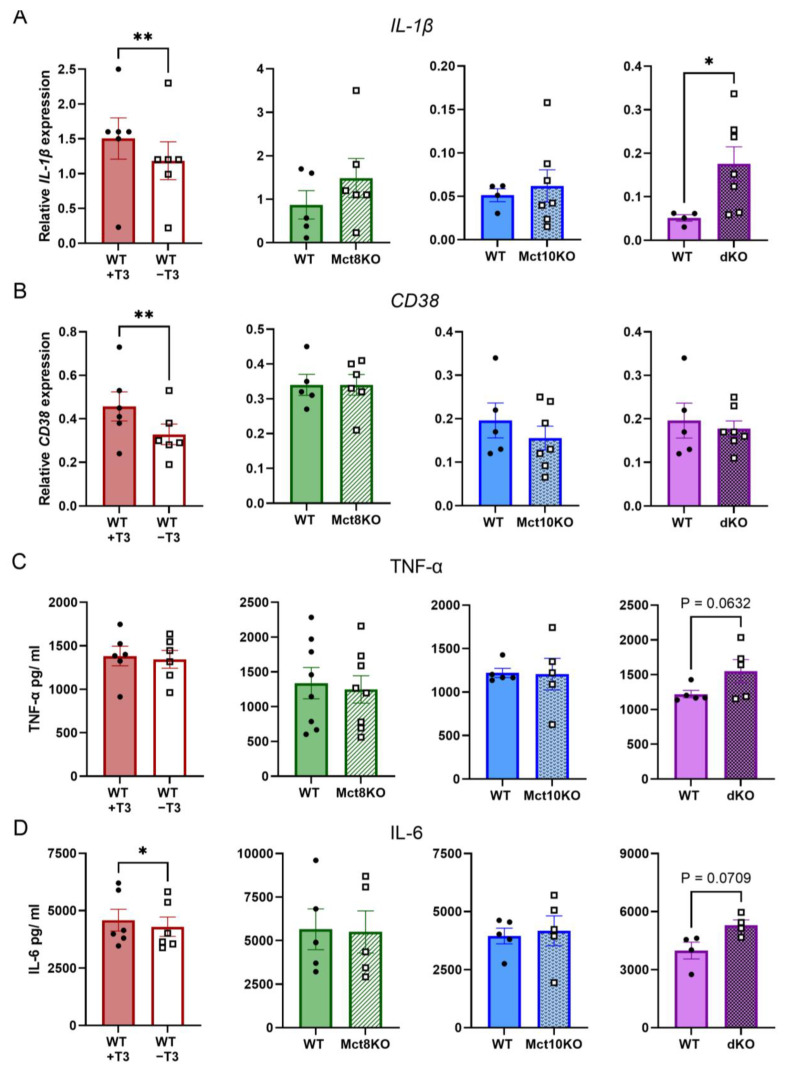
Expression of proinflammatory genes and secretion of cytokines in M1 WT, Mct8 KO, Mct10 KO and Mct10/Mct8 dKO (dKO) BMDMs. Colored bars with closed circles represent WT BMDMs treated with 10 nM T3 or WT BMDMs and open or patterned bars with open squares represent WT BMDMs treated with T3-depleted medium or Mct8 KO, Mct10 KO and Mct10/Mct8 dKO BMDMs. Using qPCR, the expression of *IL-1β* (**A**) and *CD38* (**B**) was measured. Mean values ± SEM are depicted. Differences between groups were analyzed using paired (T3-depleted or stimulated BMDMs) or unpaired (WT, Mct8 KO, Mct10 KO and Mct10/Mct8 dKO BMDMs) two-tailed Student’s *t*-test: * *p* < 0.05, ** *p* < 0.01. With ELISA the secretion of TNF-α (**C**) and IL-6 (**D**) was studied. Mean values ± SEM are depicted. Differences between groups were analyzed using a paired two-tailed Student’s *t*-test: * *p* < 0.05.

*Il1b* mRNA expression was reduced in M1 T3-depleted BMDMs compared with the cells that were cultured in the presence of T3. Notably, *Il1b* mRNA expression increased in M1-polarized BMDMs of Mct10/Mct8 dKO mice when compared with their respective controls (Figure 5A). Depletion of T3 also resulted in the transcriptional reduction of the M1 gene *CD38*, while the expression remained unchanged in transporter KOs (Figure 5B). mRNA expression of the proinflammatory genes *Fpr2* and *Gpr18* was also measured and was unchanged in all conditions (Figure S4). TNF-α secretion did not differ in all conditions, although an increasing trend was visible in Mct10/Mct8 dKO M1 BMDMs (*p* = 0.063, Figure 5C). IL-6 secretion was decreased in T3-depleted M1 BMDMs, while a tendency to increased secretion (*p* = 0.071) can be observed in macrophages derived from Mct10/Mct8 dKO mice (Figure 5D). These results further support the observation that M1 polarization is impaired in T3-depleted BMDMs, but stimulated in Mct10/Mct8 dKO BMDMs.

### 2.5. M2 Membrane Marker Expression of CD206 in BMDMs

**Figure 6:**
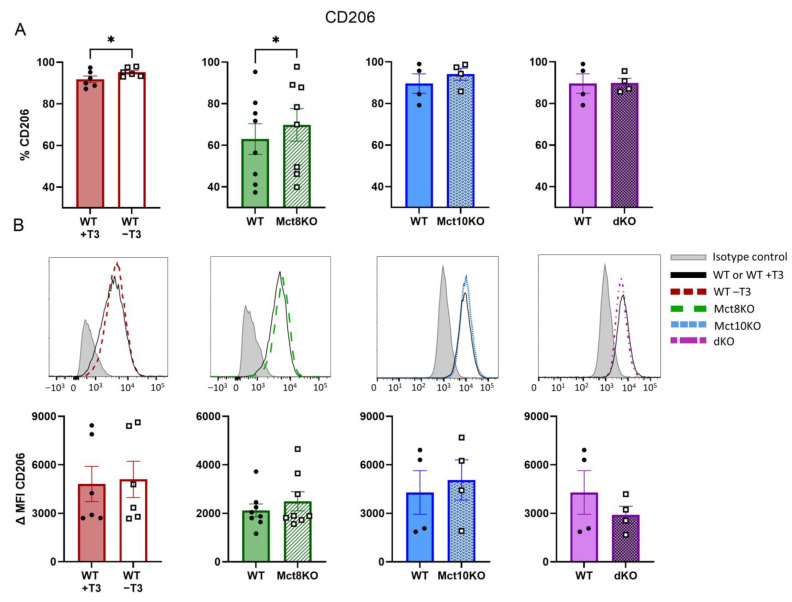
Expression of immunomodulatory surface marker CD206 as measured by flow cytometry in M2-polarized WT, Mct8 KO, Mct10 KO and Mct10/Mct8 dKO (dKO) BMDMs. Colored bars with closed circles represent WT BMDMs treated with 10 nM T3 or WT BMDMs and open or patterned bars with open squares represent WT BMDMs treated with T3-depleted medium or Mct8 KO, Mct10 KO and Mct10/Mct8 dKO BMDMs. The percentage of cells expressing CD206 (**A**) and representative histograms with accompanying quantified delta median fluorescence intensities (ΔMFIs, **B**) are shown. Mean values ± SEM are depicted. Differences between groups were analyzed using a paired two-tailed Student’s *t*-test: * *p* < 0.05.

Similar to M1 macrophages, M2 macrophages can also be identified by specific markers, which include CD206 [15,16]. To determine the effect of T3 and the lack of Mct8 and Mct10 on M2 polarization, CD206 expression was measured using flow cytometry. The percentage of cells expressing CD206 increased in both T3-depleted and Mct8 KO BMDMs, while expression levels determined by the ΔMFIs were unchanged in all BMDMs (Figure 6A,B). These results suggest that more macrophages polarize toward an M2 phenotype in the absence of T3 or Mct8 compared with corresponding control cells.

### 2.6. mRNA Expression of M2 Markers in BMDMs

**Figure 7:**
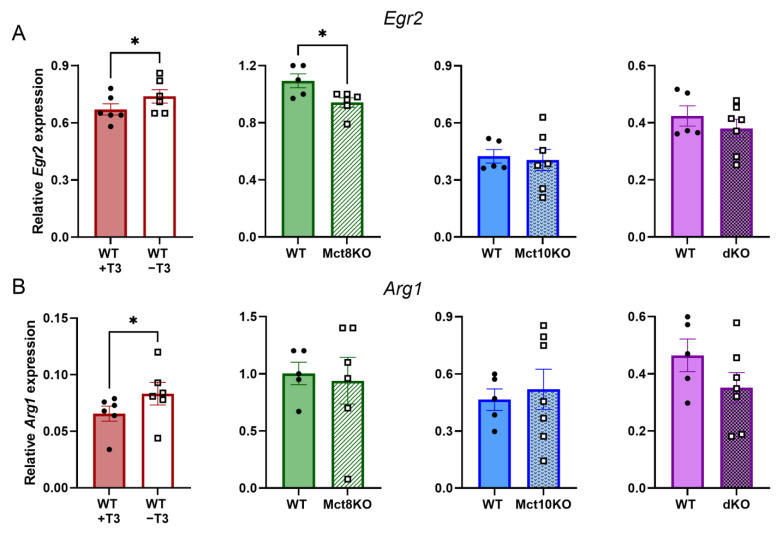
Expression of immunomodulatory genes in M2 WT, Mct8 KO, Mct10 KO and Mct10/Mct8 dKO (dKO) BMDMs. Colored bars with closed circles represent WT BMDMs treated with 10 nM T3 or WT BMDMs and open or patterned bars with open squares represent WT BMDMs treated with T3-depleted medium or Mct8 KO, Mct10 KO and Mct10/Mct8 dKO BMDMs. Using qPCR, the expression of *Egr2* (**A**) and *Arg1* (**B**) was investigated. Mean values ± SEM are depicted. Differences between groups were analyzed using paired (T3-depleted or stimulated BMDMs) or unpaired (WT, Mct8 KO, Mct10 KO and Mct10/Mct8 dKO BMDMs) two-tailed Student’s *t*-test: * *p* < 0.05.

Next, we assessed the expression of immunomodulatory markers *Egr2* and *Arg1* in M2 BMDMs. In cells depleted of T3, both *Egr2* and *Arg1* mRNA expression increased, while M2 BMDMs of Mct8 KO mice showed reduced *Egr2* expression (Figure 7A,B). Expression of *Egr2* and *Arg1* in M2 BMDMS of Mct10 KO and Mct10/Mct8 dKO mice did not change compared with WT cells. The immunomodulatory genes *IL-10* and *c-Myc* were measured as well, and expression was unaltered in all BMDMs (Figure S5). In accordance with the results mentioned above, these results indicate that depletion of T3 in murine macrophages stimulates immunomodulatory polarization. Contradictory to the previous results, a lack of the Mct8 transporter partially reduces this phenotype.

## 3. Discussion

This study examines the impact of T3 on macrophage polarization, with a particular focus on the involvement of TH transporters Mct8 and Mct10. These transporters play a crucial role in determining cellular TH availability. By utilizing Mct8 KO, Mct10 KO and Mct10/Mct8 dKO mice, we selectively evaluated the specific function of these transporters in BMDMs. Not only are macrophages crucial in the first line of defense against infection, but unbalanced macrophage polarization has been associated with many pathologies such as neurodegeneration, atherosclerosis and fibrosis [7,17,18]. Multiple studies, including this one, support the regulatory role of TH in macrophage polarization, highlighting the physiological significance of TH in macrophage function.

A decrease in *Klf9* expression was observed in BMDMs lacking Mct10, but not in Mct8 KO BMDMs. This indicates reduced availability of T3 in Mct10 KO macrophages, suggesting that Mct10 is a more important TH transporter than Mct8 in murine macrophages. This is in line with previously reported mRNA expression data [21,26]. Mct10 plays an essential role in the efflux of TH in the liver and kidneys [25]. Our findings, however, demonstrate that Mct10 is also significant for influx in macrophages, since the absence of Mct10 reduces intracellular T3 availability. We identified Lat1 and Lat2 as additional transporters expressed on macrophages, although it remains unclear what their contribution to TH transport in macrophages is. It is unknown which other TH transporters are expressed on macrophages as this area has not been fully explored [24]. 

We showed that T3 deficiency during polarization led to a decrease in most proinflammatory markers in M1 BMDMs while increasing the majority of immunomodulatory markers in M2 BMDMs. This outcome is in line with earlier findings where polarized BMDMs depleted of T3 showed reduced M1 markers, as well as amplified M2 markers compared with BMDMs stimulated with supraphysiological amounts of T3 (500 nM) [19]. Even with higher physiological T3 levels (10 nM), the impact persists. The secretion of IL-6, a proinflammatory cytokine, was reduced in T3-depleted M1 BMDMs. Another study also reported on decreased proinflammatory markers in T3-deficient macrophages, although IL-6 was unaffected in different macrophage cell lines and primary peritoneal macrophages [20]. In a study where BMDMs were stimulated with IFN-y, which elicits a proinflammatory response, phosphorylated STAT-1 and JAK-2 were more abundant in T3-depleted BMDMs [27]. The JAK/STAT-1 pathway is an inducer of the M1 phenotype [28,29]. This is in contrast with our data, as we found decreased proinflammatory markers in M1 BMDMs without T3. It is important to note, however, that in this study BMDMs were only stimulated with IFN-y for 2 h, whereas in the current report BMDMs were stimulated with a combination of LPS and IFN-y for 24 h [27].

The role of T3 signaling in macrophage polarization has been studied in various settings, targeting both type 2 deiodinase (D2), the T3-activating enzyme, and TRα, the dominant TR in macrophages [26]. Knockdown of D2, which reduces intracellular T3 availability, resulted in a decrease in proinflammatory marker expression and impaired phagocytosis in a macrophage cell line stimulated with LPS [26]. Similarly, D2 KO in M1 BMDMs partially reduced cytokine expression and phagocytosis capacity [21]. In the same study, knockdown of TRα in a macrophage cell line led to reduced M1 and elevated M2 markers [21], which is in agreement with the results we obtained after T3 depletion. Conversely, another study found that mRNA expression of proinflammatory markers increased in TRα KO inflammatory macrophages [30]. These macrophages were directly taken from mice with inflammation of the kidney instead of in vitro stimulation of BMDMs, which could rationalize the discrepancy between these studies [21,30].

This study focuses on the role of TH transporters, important mediators in T3 signaling, in macrophage function. The objective of this study was to investigate the polarization capability of Mct8 KO, Mct10 KO and Mct10/Mct8 dKO BMDMs in the M1 or M2 phenotype in a condition representing the physiological situation. To achieve this, the culture medium was supplemented with 10% FBS, resulting in detectable total T4 and T3 concentrations [31]. Despite the fact that the concentrations reached in the culture medium are lower than the physiological levels in WT mice (approx. 70 nM T4 and 0,8 nM T3), a clear reduction in the expression of *Klf9* (a T3-responsive gene) was observed in Mct10 KO and Mct10/Mct8 dKO BMDMs compared with WT mice, indicating that T3 transport is indeed reduced. We therefore feel that our experimental setting can be used to test those BMDMs adequately.

Deleting the Mct8 transporter in BMDMs did not impact intracellular T3 levels, indicating that the transporter is not crucial for TH influx or efflux in these cells. This is in agreement with the observation that mRNA expression of Mct8 in BMDMs is low in both previous reports and the current study [21,26]. M1 polarization was unaffected by Mct8 deletion. Contrary to our expectations, M2 polarization was affected, as expression of the immunomodulatory surface marker CD206 increased. Contrarily, expression of the M2 mRNA marker *Egr2* decreased. It is tempting to speculate that the changes are due to other molecules rather than TH, although no other substances have been identified as being transported by Mct8 apart from TH. A recent study in mice with a dKO of the Mct8 and Oatp1c1 transporters identified many proteins that showed altered expression profiles compared with WT mice [32]. Not all of these proteins were associated with TH signaling, suggesting molecules other than TH are transported and exert an effect. However, the dKO of both Mct8 and Oatp1c1 makes it difficult to determine the role of the respective transporters individually [24]. In patients with MCT8 deficiency, the absence of TH transport into the brain leads to delayed neurological development. There are no reports suggesting that MCT8 deficiency affects the immune system in humans. Our findings, however, suggest that macrophage function may be affected, although the potential clinical implications remain uncertain.

In contrast to Mct8 KO BMDMs, Mct10 KO BMDMs showed a decrease in intracellular T3 availability as *Klf9* mRNA expression was lowered, albeit without any impact on the expression of polarization markers. This outcome is remarkable, since we expected that the Mct10 KO BMDMs would exhibit a comparable response to the BMDMs depleted of T3, given the decrease in *Klf9* expression in both cases. One potential explanation for this is that Mct10 has the ability to transport molecules other than TH, such as the precursor of dopamine, L-dopa [24]. Dopamine is suggested to have an immunomodulatory effect in macrophages [33,34,35,36]. It is possible that when Mct10 is absent in macrophages, both TH and dopamine transport are affected. The lack of the immunomodulatory effect of dopamine could potentially counteract the lack of the proinflammatory influence of T3. Macrophages, however, also express dopamine transporters, and the significance of Mct10 in dopamine transport in these cells is unknown. Further investigation is required to examine the interplay of TH and dopamine in macrophages and what the importance of Mct10 is in this.

It is noteworthy that a dKO of the Mct8 and Mct10 transporters confirmed reduced T3 availability, similar to BMDMs lacking T3 and Mct10 KO BMDMs, but with mainly increased M1 markers and unchanged M2 markers, contrary to T3-depleted BMDMs. This suggests that M2 polarization is not disrupted by dKO of the Mct8 and Mct10 transporters, while it moderately affects M1 polarization. Furthermore, the proinflammatory markers affected in the dKO BMDMs differed partially from those altered in T3-deficient BMDMs. An absence of both Mct8 and Mct10 might not only dysregulate TH transport, but could also inhibit influx or efflux of other potential molecules that are transported by these transporters. It is also possible that after removing both Mct8 and Mct10, other transporters may compensate for their absence. Expression of the transporters Lat1 and Lat2 was unchanged in BMDMs of all KOs. However, this does not rule out that changes in other transporters may occur due to the loss of Mct8 and Mct10 that might affect transport of molecules other than TH that play a role in polarization. Alternatively, the systemic absence of Mct8 KO, Mct10 KO and Mct10/Mct8 dKO may result in changes in other organ systems that are able to alter immune function indirectly. As there are no known patients with deficiencies of MCT10 or both MCT8 and MCT10, the clinical implications of the defects and the role of other TH transporters on macrophage polarization remain unknown.

In conclusion, T3 deficiency decreases M1 markers in proinflammatory BMDMs and induces M2 markers in immunomodulatory BMDMs, suggesting a proinflammatory effect of T3 in these cells. In contrast, deletion of Mct10, the main TH transporter in macrophages, results in impaired T3 signaling while macrophage polarization is unaffected. Further research is required to determine the causes and potential influence of other molecules that are transported by Mct8 and Mct10 on macrophage polarization. The effects of T3 and TH transporters on functional aspects of polarized macrophages also deserve additional investigation. Understanding the mechanisms involved in macrophage polarization is necessary as imbalanced macrophage polarization can lead to a wide range of diseases.

## 4. Materials and Methods

### 4.1. Cell Culture

Frozen bone marrow from male and female Mct8 KO, Mct10 KO, Mct10/Mct8 dKO and corresponding WT C57BL/6 mice was kindly provided by Heike Heuer, University Duisburg, Essen [25,37,38,39]. WT, KO and dKO mice were obtained and genotyped as previously reported [25,39]. Mice were housed at 22 °C with a 12 h light and 12 h dark cycle and had access to ad libitum regular chow and water. Mice were aged 3.5 to 4.5 months when they were sacrificed by cervical dislocation. Bone marrow was isolated from the femur and tibia of the mice by rinsing the inside of the bone with sterile phosphate-buffered saline (PBS). The isolated cells were frozen in 1 mL fetal bovine serum (FBS) containing 10% [volume-to-volume ratio (*v*/*v*)] dimethyl sulfoxide (DMSO). The number of male and female bone marrow samples was similar in all experiments and matched at the start of culturing. To obtain bone marrow-derived macrophages (BMDMs), frozen cells from the bone marrow were thawed and washed in Dulbecco’s modified Eagle medium (DMEM)/F12 medium (Gibco, Paisley, Scotland) supplemented with 10% (*v*/*v*) FBS and 1% (*v*/*v*) penicillin–streptomycin–neomycin (PSN), 1% (*v*/*v*) GlutaMAX (Gibco, Paisley, Scotland) and 15 or 20% (*v*/*v*) L929-conditioned medium (16). Culture medium supplemented with 10% FBS generally contains 8 nM T4 and 0.25 nM T3 [31]. L929 cells secrete macrophage colony stimulating factor (M-CSF) and other proteins [40]. Approximately 10 × 10^6^ cells were plated in non-tissue culture-treated 150 × 15 mm petri dishes in 30 mL medium and cultured at 37 °C with 5% CO_2_. An additional 15 mL of DMEM/F12 with L929 was added on day 3. At day 7 of the culture, the BMDMs were harvested using TrypLE Express Enzyme (Gibco, Paisley, Scotland) and transferred to 6-well plates (1.5 × 10^6^ cells/well) in DMEM/F12 without L929-conditioned medium. After 24 h, the BMDMs were stimulated with either 20 ng/mL lipopolysaccharide (LPS, Invitrogen, Darmstadt, Germany) and 20 ng/mL interferon (IFN)-γ (Gibco, Paisley, Scotland) for M1 polarization or 20 ng/mL interleukin (IL)-4 (Invitrogen, Darmstadt, Germany) for M2 polarization. As a control, unstimulated BMDMs were included, referred to as M0 macrophages. Polarization of BMDM lasted 24 h in all experiments. To study the effect of T3 depletion on macrophage function, additional WT BMDMs were polarized as described above and cultured in DMEM/F12 without L929 and with 10% charcoal-stripped FBS/vehicle (T3-depleted) or 10 nM T3 (physiological concentration of T3). 

### 4.2. Flow Cytometry

Surface marker expression of the BMDMs was measured using flow cytometry. Samples were incubated with Fc block (eBioscience, Hatfield, U.K.) prior to antibody staining (Table 1). To correct for background fluorescence, isotype control antibodies were matched to antibodies used for staining. Unstained controls were always measured, and compensation was set using OneComp eBeads (Invitrogen, Darmstadt, Germany). Fluorescence was quantified on a FACSCanto II (BD Biosciences, Eysins, Switzerland) or FACSSymphony A1 (BD Biosciences, Eysins, Switzerland) and analyzed with FlowJo (v. 10) software. Isotype controls were used to set gates. The gating strategy is detailed in Figure S6. Macrophages were identified as CD11b+/F4/80+ cells. To determine the expression of the cell surface markers, the median fluorescence intensity (MFI) of each marker within the CD11b+/F4/80+ cell population was measured. The MFI of the isotype controls was then subtracted from the MFI of the markers of interest to account for background fluorescence, resulting in the ΔMFI. The baseline expression of the different polarization surface markers on WT M0, M1 and M2 BMDMs is depicted in Figure S7.

### 4.3. RNA Isolation and Quantitative Real-Time PCR

RNA was isolated with the Roche High Pure RNA isolation kit (Roche, Mannheim Germany) according to the manufacturer’s instructions. The RNA concentration was quantified by the DeNovix nanodrop. For cDNA synthesis, the Transcriptor First Strand cDNA synthesis Kit (Roche, Mannheim Germany) was used according to the manufacturer’s instructions with equal amounts of RNA per experiment. Multiple randomly chosen samples were additionally processed without reverse transcriptase during cDNA synthesis to check for genomic DNA contamination. SensiFAST SYBR No-ROX (Bioline, London, England) and the LightCycler480 (Roche, Mannheim Germany) were used to perform quantitative PCR. Primer sequences are detailed in Table 2. The data were analyzed with LC480 conversion (v. 2014.1) and LinRegPCR (v. 2021.1) software and results were efficiency-corrected [41]. The geometric mean of three reference genes (*Ppib*, *Rplp0* and *Ubc*) was used to normalize expression levels of mRNA in the samples, therefore showing relative expression values.

### 4.4. Cytokine Measurement

The levels of IL-6 and TNF-α, proinflammatory cytokines, in the supernatant of polarized M1 macrophages were measured using the IL-6 Mouse Uncoated ELISA Kit (Invitrogen, Darmstadt, Germany) and TNF-α Mouse Uncoated ELISA Kit (Invitrogen, Darmstadt, Germany) according to the manufacturer’s instructions. Samples were diluted 1:19 in ELISAPOT Diluent from the respective kit and run in duplicate using the Varioskan Flash (Thermo Scientific, Breda, the Netherlands) with the software SkanIt RE (v. 2.4.5). The results were corrected for the number of cells in the 6-well plate.

### 4.5. Statistical Analysis

For statistical analysis, GraphPad Prism 9 was used. Statistical significance was tested with a two-tailed unpaired or paired Student’s *t*-test where results with *p*-values < 0.05 were considered statistically significant. All experiments where WT BMDM were stimulated with T3- or T3-depleted serum were statistically tested with a paired *t*-test, as both conditions derived from the same macrophage culture. Flow cytometry or ELISA results for WT, Mct8 KO, Mct10 KO and Mct10/Mct8 dKO BMDMs were also analyzed with a paired *t*-test, as each KO culture has a corresponding WT in the same culture setup. In this way, day to day variation is excluded. qPCR data of these BMDMs were tested with an unpaired *t*-test, as measurements were performed for all samples simultaneously and normalized by reference genes. Data are presented as mean ± standard error of the mean (SEM). Any outliers were identified with Dixon’s Q-test and excluded from the data [43].

## Figures and Tables

**Table 1:** Antibodies for flow cytometry.

Surface Marker Target	Fluorescent Conjugate	Species Raised In; Mono- or Polyclonal	Clone	Catalog Number	Manufacturer	Dilution
F4/80	APC-eFluor780	Rat IgG2a, κ; Monoclonal	BMB	47-4801-82	Invitrogen, Darmstadt, Germany	1:100
CD11b	FITC	Rat IgG2b, κ; Monoclonal	M1/70	11-0112-41	Invitrogen, Darmstadt, Germany	1:100
CD206	APC	Rat IgG2a, κ; Monoclonal	C068C2	141707	Biolegend, Amsterdam, the Netherlands	1:100
CD86	APC	Rat IgG2a, κ; Monoclonal	GL1	17-0862-81	Invitrogen, Darmstadt, Germany	1:200
CD80	PE	Hamster IgG2, κ; Monoclonal	16-10A1	561955	BD Bioscience, Eysins, Switzerland	1:400
CD16/CD32 (Fc-block)	-	Rat IgG2a, λ; Monoclonal	93	14-0161-85	Invitrogen, Darmstadt, Germany	1:50

**Table 2:** Primer sequences for qPCR.

Gene	Protein	Forward (5′-3′)	Reverse (5′-3′)	Amplicon Length	Source	Accession Number
*Ppib*	Cyclophilin-B	GAGACTTCACCAGGGG	CTGTCTGTCTTGGTGCTCTCC	253	[21]	NM_011149.2
*Rplp0*	Rplp0	GGCCCTGCACTCTCGCTTTC	TGCCAGGACGCGCTTGT	124	[21]	NM_007475.5
*Ubc*	Ubc	AGCCCAGTGTTACCACCAAG	CTAAGACACCTCCCCCATCA	118	[26]	NM_019639.4
*Slc16a10*	Mct10	GATGAACATGGCCTCCCAACA	CCTTGAAGTGAGTCTGGCTGG	164	Newly designed	NM_001114332.1
*Slc16a2*	Mct8	GGTGGAGTCACTGTCCTGTC	GCACACTTATTCTGCCCCCT	180	Newly designed	NM_009197.2
*Slc7a5*	Lat1	AAGGGCAGGGATTCATGGTG	GTAGGGGTGTCTTTCAGGGC	188	Newly designed	NM_011404.3
*Slc7a8*	Lat2	GCAAGAAAGTACCTGAGCACG	AACAGATCGCCTCCACCTTC	106	Newly designed	NM_016972.2
*Klf9*	Klf9	CCACCGAATCTGGGTCGAG	TCCGAGCGCGAGAACTTTT	265	Newly designed	NM_010638.5
*Il1b*	IL-1β	TTGACGGACCCCAAAAGATG	AGAAGGTGCTCATGTCCTCA	204	[42]	NM_008361.4
*CD38*	CD38	ACTGGAGAGCCTACCACGAA	AGTGGGGCGTAGTCTTCTCT	179	Newly designed	NM_007646.6
*Fpr2*	Fpr2	ATTTACACCACAGGAACCGAAGA	TGATGGAGACAACCACCATTGA	166	Newly designed	NM_008039.2
*Gpr18*	Gpr18	ATCTGCTTTGCCGTCCTGAT	ACTGCGAAGGTAATTGCGGT	180	Newly designed	NM_182806.2
*Egr2*	Egr2	GGGTCTGCATGTGTACAGGA	AAACAAATCAGCGGCAGTGAC	209	Newly designed	NM_010118.3
*Arg1*	Arg1	CAGCACTGAGGAAAGCTGGT	CAGACCGTGGGTTCTTCACA	132	[21]	NM_007482.3
*Il10*	IL-10	ATGCAGGACTTTAAGGGTTACTTG	TAGACACCTTGGTCTTGGAGCTTA	254	[42]	NM_010548.2
*c-Myc*	c-Myc	GGAACGTCAGAGGAGGAACG	TGCTCGTCTGCTTGAATGGA	147	Newly designed	NM_010849.4

## Data Availability

The data presented in this study are available on request from the corresponding author.

## Supplementary Materials

The following supporting information can be downloaded at https://www.mdpi.com/article/10.3390/ijms25042111/s1.
