# Supplementary material for: The Differential Effect of a Shortage of Thyroid Hormone Compared with Knockout of Thyroid Hormone Transporters Mct8 and Mct10 on Murine Macrophage Polarization
Source: Int J Mol Sci. 2024 Feb 9;25(4):2111. doi: 10.3390/ijms25042111 (PMC10889717; doi:10.3390/ijms25042111)
Supplement: Supplementary file 1 [file ijms-25-02111-s001.zip › ijms-2813949-supplementary/IJMS-2813949-proofread-S1-5.pdf]

Supplemental results

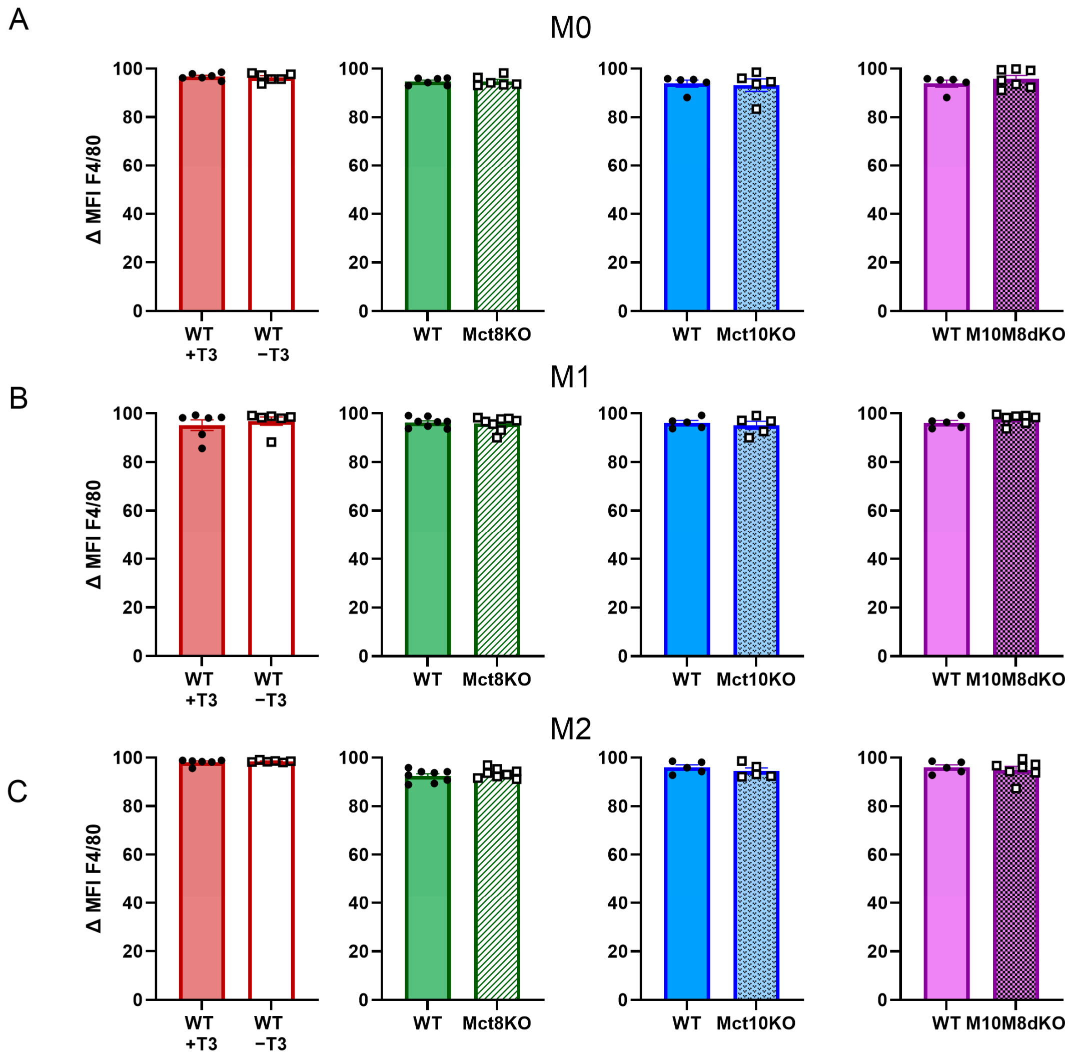

Figure S1. Percentage of cells expressing the macrophage surface marker F4/80 as measured by flow cytometry in M0, M1 and M2 WT, Mct8 KO, Mct10 KO and Mct10/Mct8 dKO (dKO) BMDMs. Colored bars with closed circles represent WT BMDMs treated with 10 nM T3 or WT BMDMs and open or patterned bars with open squares represent WT BMDMs treated with T3-depleted medium or Mct8 KO, Mct10 KO and Mct10/Mct8 dKO BMDMs. Mean values ± SEM are depicted. Differences between groups were analyzed using a paired two-tailed Student's *t*-test.

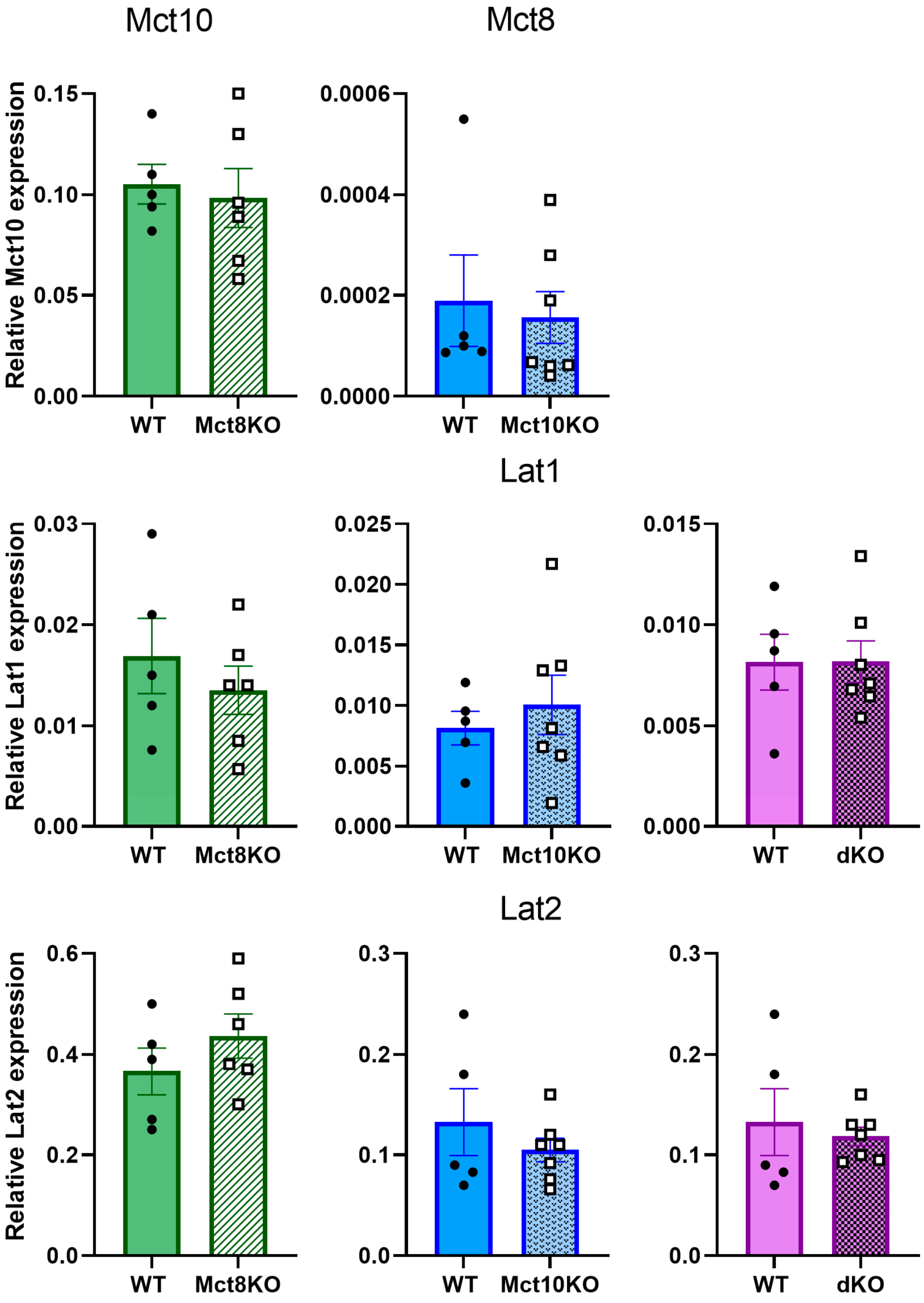

Figure S2. Relative mRNA expression of Mct8, Mct10, Lat1 and Lat2 in WT, Mct8 KO, Mct10 KO and Mct10/Mct8 dKO (dKO) M1 BMDMs. Colored bars with closed circles represent WT BMDMs and patterned bars with open squares represent Mct8 KO, Mct10 KO and Mct10/Mct8 dKO BMDMs. Mean values ± SEM are depicted. Differences between groups were analyzed using unpaired two-tailed Student's *t*-test.

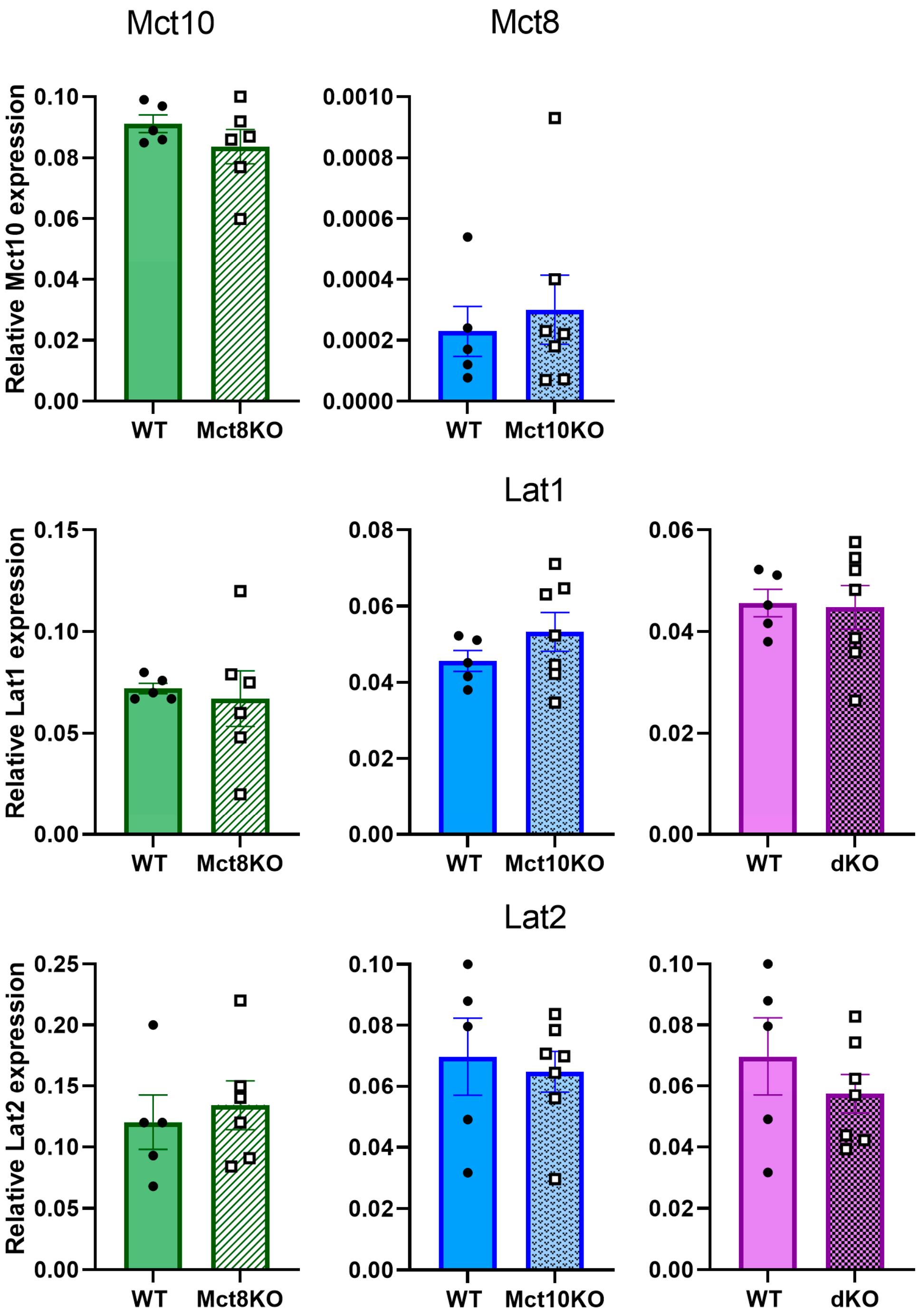

Figure S3. Relative mRNA expression of Mct8, Mct10, Lat1 and Lat2 in WT, Mct8 KO, Mct10 KO and Mct10/Mct8 dKO (dKO) M2 BMDMs. Colored bars with closed circles represent WT BMDMs and patterned bars with open squares represent Mct8 KO, Mct10 KO and Mct10/Mct8 dKO BMDMs. Mean values ± SEM are depicted. Differences between groups were analyzed using unpaired two-tailed Student's *t*-test.

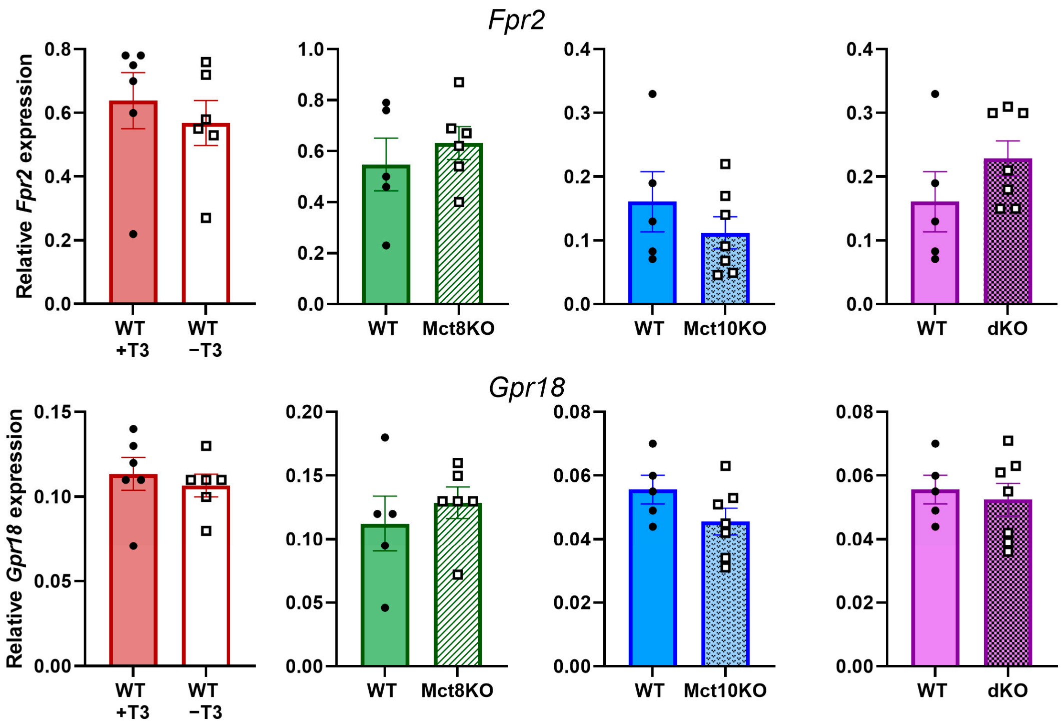

Figure S4. Expression of pro-inflammatory genes in M1 WT, Mct8 KO, Mct10 KO and Mct10/Mct8 dKO (dKO) BMDMs. Colored bars with closed circles represent WT BMDMs treated with 10 nM T3 or WT BMDMs and open or patterned bars with open squares represent WT BMDMs treated with T3-depleted medium or Mct8 KO, Mct10 KO and Mct10/Mct8 dKO BMDMs. Using qPCR, the expression of *Fpr2* and *Gpr18* was investigated. Mean values ± SEM are depicted. Differences between groups were analyzed using unpaired two-tailed Student's *t*-test.

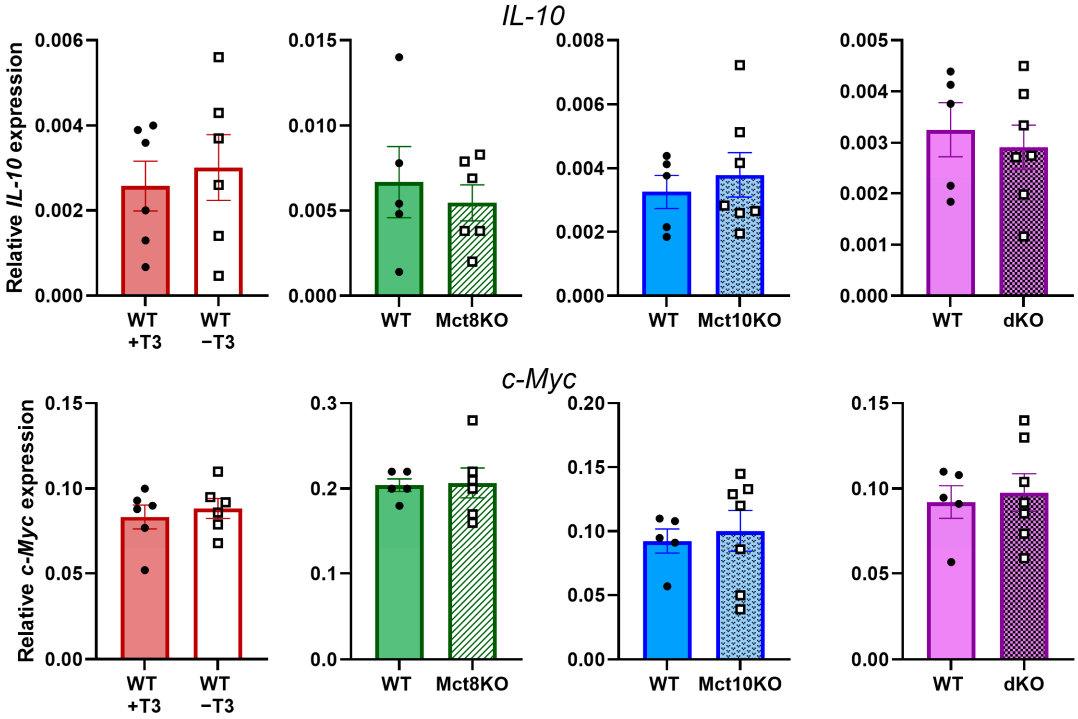

Figure S5. Expression of immunomodulatory genes in M2 WT, Mct8 KO, Mct10 KO and Mct10/Mct8 dKO (dKO) BMDMs. Colored bars with closed circles represent WT BMDMs treated with 10 nM T3 or WT BMDMs and open or patterned bars with open squares represent WT BMDMs treated with T3-depleted medium or Mct8 KO, Mct10 KO and Mct10/Mct8 dKO BMDMs. Using qPCR, the expression of *IL-10* and *c-Myc* was investigated. Mean values ± SEM are depicted. Differences between groups were analyzed using unpaired two-tailed Student's *t*-test.
