# Supplementary material for: The Differential Effect of a Shortage of Thyroid Hormone Compared with Knockout of Thyroid Hormone Transporters Mct8 and Mct10 on Murine Macrophage Polarization
Source: Int J Mol Sci. 2024 Feb 9;25(4):2111. doi: 10.3390/ijms25042111 (PMC10889717; doi:10.3390/ijms25042111)
Supplement: Supplementary file 1 [file ijms-25-02111-s001.zip › ijms-2813949-supplementary/ijms-2813949-proofread-S6.pdf]

Supplemental methods

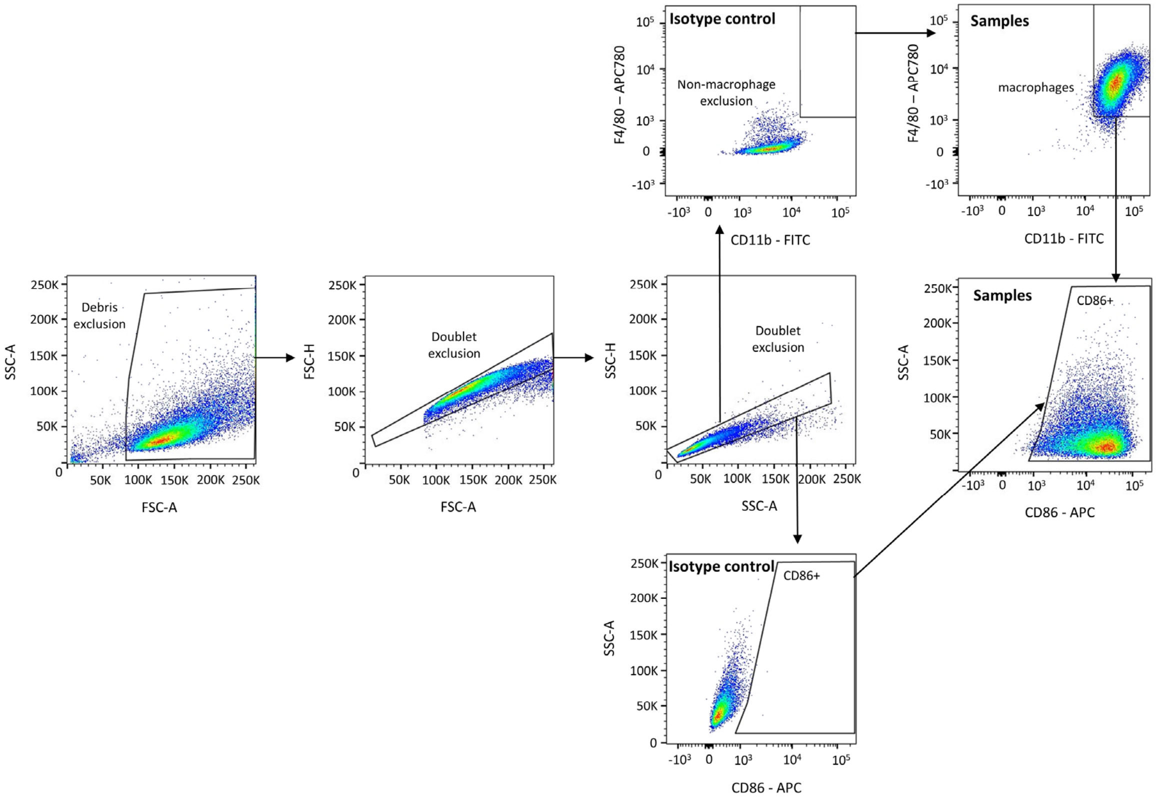

Figure S6. Gating strategy of BMDMs in FlowJo. Debris and doublets were excluded before selecting macrophages using the markers CD11b and F4/80. Gates were set with isotype controls for macrophage (CD11b+/F4/80+) and marker selection. Samples that were gated as macrophages and positive for the respective marker were analyzed. For all markers the gating strategy was similar, but with the respective isotype control.
