# Supplementary material for: The Differential Effect of a Shortage of Thyroid Hormone Compared with Knockout of Thyroid Hormone Transporters Mct8 and Mct10 on Murine Macrophage Polarization
Source: Int J Mol Sci. 2024 Feb 9;25(4):2111. doi: 10.3390/ijms25042111 (PMC10889717; doi:10.3390/ijms25042111)
Supplement: Supplementary file 1 [file ijms-25-02111-s001.zip › ijms-2813949-supplementary/ijms-2813949-proofread-S7.pdf]

Supplemental methods

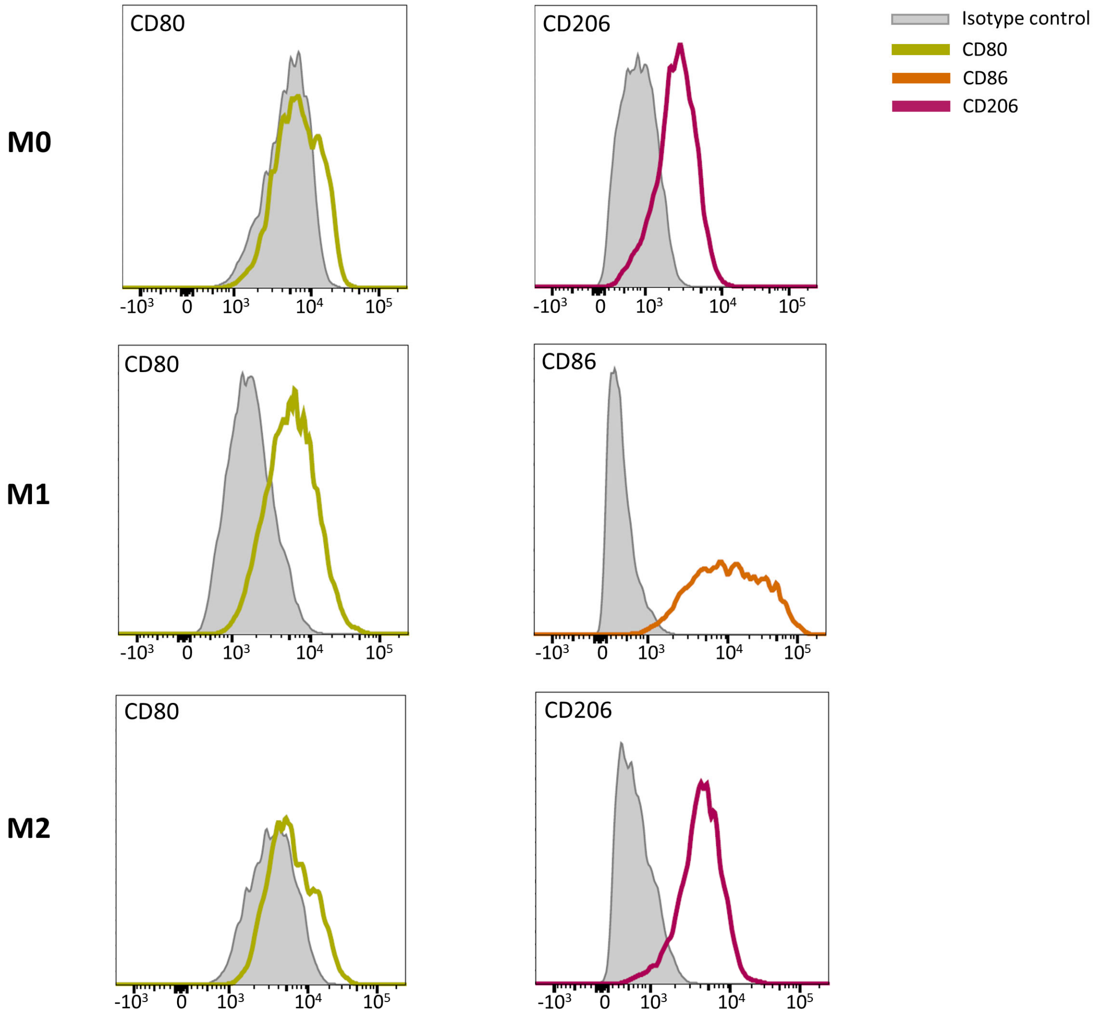

Figure S7. Expression of CD80, CD86 and CD206 on WT M0, M1 and M2 BMDMs. Grey histograms represent the isotype control and the colored line represents the measured surface marker.
